# Discriminative ability of the generic and condition specific Oral Impact on Daily Performance (OIDP) among adolescents with and without hypodontia

**DOI:** 10.1186/1472-6831-14-57

**Published:** 2014-05-22

**Authors:** Christina L Hvaring, Kari Birkeland, Anne N Åstrøm

**Affiliations:** 1 Department of Orthodontics, Faculty of Dentistry, University of Oslo, Oslo, Norway; 2 Department of Clinical Dentistry, Community Dentistry, University of Bergen, Bergen, Norway

**Keywords:** Hypodontia, Tooth agenesis, Missing teeth, Malocclusion, Oral health related quality of life, OHRQoL, Oral impact on daily performance, OIDP, Adolescent, Child

## Abstract

**Background:**

The aims of this study were to (1) investigate to what extent the generic and condition specific (CS) forms of the oral impact of daily performance (OIDP) inventory discriminate between a group of patients with hypodontia and a group of patients having malocclusion, (2) assess the association of the generic and CS OIDP with severity and localisation of hypodontia, whilst adjusting for patients’ age and sex.

**Methods:**

A total of 163 patients aged 10–17 years were included in a cross-sectional study. Two groups were investigated: 62 patients with non-syndromic hypodontia and 101 non-hypodontia patients. Both groups had a malocclusion of similar treatment need. All patients underwent a clinical and radiographic examination and completed a Norwegian version of the generic and the CS OIDP inventory. CS scores were established for impacts attributed to hypodontia.

**Results:**

The mean number of missing teeth in the hypodontia group was 6.2. The prevalence of generic and CS oral impacts in the hypodontia group were 64% and 30%, and the corresponding rates in the non-hypodontia group were 62% and 10%. The generic OIDP did not discriminate between the two groups with respect to overall scores. The CS OIDP discriminated strongly between patients with and without hypodontia regarding problems with emotional status, showing teeth, social contact, speaking and carrying out work. Compared to the non-hypodontia group, patients with hypodontia, with severe hypodontia (≥ 6 missing teeth) and upper anterior hypodontia were respectively 3.4, 2.5 and 7.0 times more likely to report any oral impact attributed to small teeth, gaps between teeth and missing teeth.

**Conclusions:**

Hypodontia and malocclusion patients report a considerable burden of oral impacts. The CS-OIDP measure discriminated most effectively between patients with and without hypodontia and was related to severity and upper anterior localisation of hypodontia.

## Background

Hypodontia, the absence of at least one primary or permanent tooth excluding third molars, is one of the most common congenital developmental anomalies [1]. In most cases, the affection is mild, with absence of one or two teeth, but it may also involve multiple permanent teeth and represent a great clinical challenge. The prevalence of hypodontia in the Norwegian population has been reported to be 6.5% [2]. It varies, however, from 2.6 to 11.3% in epidemiological studies from Asia, Europe and North America [3]. Oligodontia, defined as the absence of six or more permanent teeth, affects less than 0.2% of the general Norwegian and Danish population [4,5]. Hypodontia can occur as an isolated condition (non-syndromic hypodontia) or be associated with a syndrome (syndromic hypodontia) [6].

Missing a large number of teeth at an early age represents a source of social and psychological distress [7]. Even though patients with hypodontia may be diagnosed early, treatment does not commence until late adolescence as dental implant restorations, when necessary, should preferably be carried out after growth has ceased. Other treatment options such as orthodontic space closure, autotransplantation and conventional or resin-bonded bridges depend on factors such as the number of missing teeth, occlusion, age, facial morphology, tooth morphology, and need for orthodontic treatment in general. The teenage years are a formative period when perception of appearance may impact on adaptation to social settings [8]. Understanding how hypodontia affects general wellbeing and oral health related quality of life (OHRQoL) provides insight into the day-to-day lives of the patients and improves the therapeutic approach. Thus, a traditional normative approach to assess oral health using only clinical measures has serious inadequacies [9]. Generic and disease specific OHRQoL measures have been developed to improve understanding of the psychosocial consequences of oral diseases upon quality of life and to complement the traditional clinical measures.

One OHRQoL measure, the Oral Impact on Daily Performance (OIDP) scale, was developed to measure oral impacts that seriously affect individuals’ daily activities [10]. This instrument has proven to be reliable and valid in cross-sectional population-based studies as well as in studies of patients with specific oral disorders [11,12]. The OIDP inventory has been translated into Norwegian, and validated in a representative sample of the Norwegian adult population [13]. It is designed as a generic or condition specific (CS) measure. While the generic inventory assesses the overall impact of oral problems simultaneously, the CS form focuses on impacts attributed to a particular disease or condition, such as hypodontia. This makes the CS instrument more sensitive to small, but clinically relevant changes in specific oral diseases [14,15]. The CS instrument may provide insight into the consequences of an untreated oral condition and is particularly important when assessing treatment need as well as prioritizing dental health care services [16].

Recently, a small number of studies have assessed the psychosocial impacts of hypodontia among children and adolescents. British studies by Laing et al. [17] and Kotecha et al. [18] compared children with hypodontia with a malocclusion control group using the child perceptions questionnaire (CPQ). Laing et al. [17] compared hypodontia patients to a malocclusion group of similar normative treatment need and found no difference in quality of life. Kotecha et al. [18] compared hypodontia patients to a malocclusion group of lesser normative treatment need and observed significantly higher oral impacts in the hypodontia group. Wong et al. [19] and Locker et al. [20] investigated quality of life using the CPQ in children with hypodontia, but without a control group. The reported prevalence of oral impacts was 100% and 80%, respectively. Locker et al. [20] compared the results to published data for other clinical groups and concluded that hypodontia patients reported higher rates of oral impacts.

Since only a few studies have focused on OHRQoL in young people with hypodontia, it seems important to increase the current knowledge in this area by examining a Norwegian population of hypodontia patients. The aims of this study were to (1) investigate to what extent the generic and CS form of the oral impact of daily performance (OIDP) inventory discriminate between a group of patients with hypodontia and a group of patients having malocclusion, (2) assess the association of the generic and CS OIDP with severity and localisation of hypodontia, whilst adjusting for patients’ age and sex. It was hypothesized that the CS OIDP would distinguish better than the generic OIDP between adolescents with and without hypodontia.

## Methods

The study was approved by The Regional Committee for Medical and Health Research Ethics and the Data Protection Official for Research. The subjects had all been referred to the Department of Orthodontics at the University of Oslo, and were consecutively recruited from January 2012 to September 2013. Two groups of patients aged 10 to 17 years were included in the study: 62 patients with non-syndromic hypodontia and 101 non-hypodontia patients with a malocclusion of similar normative treatment need, as measured by an index of orthodontic treatment need (IOTN), dental health component (DHC) of 4 or 5. The group assignment was confirmed by radiographic and clinical examination by the same investigator (CH), and the severity of hypodontia denoted. Exclusion criteria were associated medical history or craniofacial anomaly. Each patient and guardian was given written information about the study and written consent was obtained. Participants provided demographic data in terms of sex and age.

A sample size calculation was performed using the OpenEpi (Open Source Epidemiologic Statistics for Public Health) software. The required sample size for a chi-square test with a 0.05 level of significance to have 80% power to detect a 25% difference in prevalence of impacts was calculated to be 63 subjects in each group.

### Measures

Study participants completed a supervised self-administered questionnaire, containing oral health indicators and the eight item generic and CS oral impact on daily performance (OIDP) inventory. Unlike other OHRQoL indicators, the OIDP has already been translated into Norwegian and validated in a representative population sample, thus a set of normative data exists. The OIDP is simple in that it measures behavioural impacts only rather than feeling-state dimensions, and consists of only eight questions. It exists both in a child and adult version, where the child version has been used mainly among children and younger adolescents between 6 and 13 years [21,22]. For the present study the adult version of the OIDP was considered appropriate, as the study group contained a high number of adolescents, with ages up to 17 years.

The generic and CS OIDP frequency scores were obtained by asking:

“During the past six months, how often have you had problems with your teeth and mouth that caused you difficulty with: eating, speaking, cleaning your teeth, smiling, sleeping, emotional balance, study, or social contact”. The responses were scored on a 5-point scale: (0) never affected; (1) less than once a month; (2) once or twice a month; (3) once or twice a week, (4) every or nearly every day. If any impact was reported, the patient was asked to report the oral conditions they perceived as its main causes. Impacts attributed to ‘small teeth’ , ‘gaps between teeth’ and ‘missing teeth’ were considered to be condition specific impacts attributed to hypodontia.

For statistical analysis the response to each question was dichotomised into two categories: 0 = never affected (including the original category 0), and 1 = affected less than once a month or more often (including the original categories 1–4). The OIDP simple count score (SC) was constructed by adding these dichotomised scores for each of the eight questions. For assessing prevalence of generic impacts, OIDP SC score was itself dichotomised as 0 = no daily performance affected and 1 = at least one daily performance affected (OIDP > 0). In addition, the OIDP additive score (ADD) was constructed by adding the eight OIDP items as scored originally, indicating overall frequency of impacts. Condition specific SC and ADD scores were computed in the same manner as the generic scores, considering only those impacts that the patient had attributed to any of the three main causes related to hypodontia.

Patients’ self-reported general oral health status was scored using the questions “How do you consider your oral health?”, “How satisfied or dissatisfied are you with the appearance of your teeth or dentures?” with five possible responses for each question: (1) very good/satisfied, (2) good/satisfied, (3) neither good/satisfied nor bad/dissatisfied, (4) bad/dissatisfied or (5) very bad/dissatisfied. For the purposes of statistical analysis, the responses were dichotomised into two categories: “good/satisfied” (including the original categories 1 and 2), and “bad/unsatisfied” (including the original categories 3, 4 and 5).

Patients’ assessment of oral health compared to other was scored using the question “Compared to others boys/girls of your age, how do you consider your own oral health?” with five possible responses: (1) much better, (2) better, (3) neither better nor worse, (4) slightly worse or (5) much worse. The responses were dichotomised into “better” (including the original categories 1 and 2), and “same or worse” (including the original categories 3, 4 and 5).

Patients’ frequency of dental attendance was scored using the question “How many times have you been to the dentist during the past 5 years?” with five possible responses: (1) at least once a year, (2) 3–4 times, (3) 1–2 times, (4) I have not been to the dentist during the past 5 years. The responses were dichotomised into “at least once a year” (including the original category 1) and “less than once a year” (including the original categories 2, 3 and 4).

### Statistical analysis

Data were analysed using the Statistical Package for Social Sciences (version 19.0; SPSS Inc., Chicago, Illinois, USA). Missing responses on single variables were not substituted, and complete case analysis was used [23]. Discriminative ability of the generic and CS OIDP was assessed by estimating differences in overall mean score and prevalence of oral impacts between clinically defined groups, through their respective effect sizes and adjusted odds ratios (ORs). Non-parametric statistics were used since the overall generic and CS OIDP scores were not normally distributed. Effect size was calculated as the mean difference between groups divided by the pooled standard deviations. The widely accepted thresholds of 0.2, 0.5 and 0.8 were used to define small, moderate, and large effect sizes [24]. Bivariate relationships were assessed using cross-tabulation, chi-square statistics and the Mann–Whitney *U* test. Internal consistency reliability was assessed using Cronbach’s alpha. Multiple logistic regression analysis was performed to calculate odds ratios (OR) with 95% confidence intervals (CI). Spearman rank correlation coefficients were calculated to evaluate the association of age and number of missing teeth, totally and anteriorly, with the OIDP score.

## Results

### Profile of the study group

A total of 163 patients participated in the study. All patients completed the questionnaire at the clinic and there were no withdrawals. The study group comprised 62 patients (27 girls and 35 boys) with hypodontia and 101 (56 girls and 45 boys) controls without hypodontia, but with malocclusion diagnoses (Table 1). The mean age of the total study group was 12.9 (SD 1.5). The mean age was 13.6 (SD 2.1) in the hypodontia group and 12.5 (SD 1.5) in the non-hypodontia group. The age profile of the two groups differed (66.1% in the hypodontia group versus 47.5% in non-hypodontia group were at age 13–17, p < 0.05). There was no difference in the sex distribution. In the non-hypodontia group, 81 patients were classified as IOTN DHC 4 and 20 patients as IOTN DHC 5. Compared to patients without hypodontia, hypodontia patients reported a higher frequency of dental attendance, rated their oral health to be worse when compared to others and were more satisfied with their dental appearance (p < 0.05). The mean number of missing teeth (absolute hypodontia) in the hypodontia group was 6.2 (1–21), and the mean relative hypodontia (number of missing teeth minus number of persisting primary teeth) was 3.1 (0–14). Of the 62 patients, 31 (50.0%) had mild hypodontia (1–5 teeth missing) and 31 (50.0%) had severe hypodontia (≥ 6 teeth missing). The prevalence of upper anterior hypodontia was 51.6% and that of any anterior hypodontia 69.4%.

**Table 1:** Sex, age and self-reported oral health indicators in participants with and without hypodontia

**Variables**	**Hypodontia**	**Non-hypodontia**	**Total**
	**n = 62 n (%)**	**n = 101 n (%)**	**n = 163 n (%)**
Sex			
Male	35 (56.5)	45 (44.6)	80 (49.1)
Female	27 (43.5)	56 (55.4)	83 (50.9)
Age			
10–12 years	21 (33.9)^*^	53 (52.5)	74 (45.4)
13–17 years	41 (66.1)	48 (47.5)	89 (54.6)
Reason for dental treatment, n (%)			
Regular check up	56 (91.8)	82 (86.3)	138 (88.5)
Pain/acute	5 (8.2)	13 (13.7)	18 (11.5)
Dental attendance last 5 years			
At least once a year	45 (72.6)^*^	53 (54.1)	98 (61.3)
Less than once a year	17 (27.4)	45 (45.9)	62 (38.8)
Self-rated oral health, n (%)			
Good	46 (75.4)	72 (72.7)	118 (73.8)
Bad	15 (24.6)	27 (27.3)	42 (26.3)
Satisfaction with dental appearance, n (%)			
Satisfied	26 (42.6)^*^	24 (24.2)	50 (31.3)
Unsatisfied	35 (57.4)	75 (75.8)	110 (68.8)
Oral health compared to others, n (%)			
Better	9 (14.8)^*^	29 (29.3)	38 (23.8)
Same or worse	52 (85.2)	70 (70.7)	122 (76.3)

The total number in the different categories does not add up to 62 and 101, respectively, owing to missing responses.

^*^p < 0.05.

As shown in Table 2, the overall mean score and prevalence of impacts for the generic OIDP in the total study group were respectively 3.3 and 63.2%. Corresponding scores for the CS OIDP were 1.1 and 17.8%.

**Table 2:** Characteristics of generic and CS OIDP scores in the total sample

**Indicator**	**Generic OIDP**	**CS OIDP**
*Mean*	3.3	1.1
SD	4.8	3.6
Min. value	0	0
Max. value	31	27
*Prevalence of impact (OIDP > 0)*		
Number of cases	103	29
Percentage of cases	63.2	17.8

### Internal consistency of the generic OIDP by clinical study group

Cronbach’s alpha for the OIDP was 0.790 in the total study group, 0.716 for the non-hypodontia group and 0.837 for the hypodontia group. For the total study group, Cronbach’s alpha if any of the items were to be deleted was lower than the original value.

### Cross sectional validity of the generic OIDP score

The prevalence of impacts and mean OIDP scores differed significantly between subjects rating their oral health as good versus bad, and between those being satisfied versus dissatisfied with their dental appearance. The prevalence of impacts amounted to 58.5% and 78.6% (p < 0.05) among participants who perceived their oral status to be good and bad, respectively. Corresponding rates for the groups being satisfied versus dissatisfied with their dental appearance was 48.0% versus 70.0% (p < 0.01). The prevalence of impacts among those rating their oral health as better than others was 55.3% versus 66.4% for those rating their oral health as same or worse than others. Multiple logistic regression revealed adjusted ORs in the range from 1.6 (95% CI 0.7-3.4) (comparative oral health) to 2.5 (95% CI 1.1-5.8) (perceived oral health) and 2.5 (95% CI 1.2-4.9) (satisfaction with appearance) (Table 3).

**Table 3:** Cross-sectional validity of generic OIDP: association of generic OIDP with age, sex and perceived oral health status

**Variables**	**Mean OIDP (SD)**	**Effect size**	**OIDP > 0, % (n)**	**Unadjusted OR (95% CI)**	**Adjusted OR (95% CI)**
Age					
10–12 years	2.6 (4.2)		56.8 (42)	1	
13–17 years	3.8 (5.2)	0.2	68.5 (61)	1.7 (0.9 – 3.2)	
Sex					
Female	3.1 (4.6)		62.7 (52)	1	
Male	3.5 (5.0)	0.1	63.7 (51)	1.0 (0.6 – 2.0)	
Perceived oral health status					
Good	2.8 (4.3)		58.5 (69)	1	1
Bad	4.8 (6.0)^**^	0.4	78.6 (33)^*^	2.6 (1.1 – 5.9)	2.5 (1.1 – 5.8)^a^
Satisfaction with dental					
appearance					
Satisfied	1.7 (3.3)		48.0 (24)	1	1
Unsatisfied	4.1 (5.3)^**^	0.5	70.0 (77)^**^	2.5 (1.3 – 5.0)	2.5 (1.2 – 4.9)^a^
Oral health compared to others					
Better	2.0 (2.9)		55.3 (21)	1	1
Same or worse	3.7 (5.3)	0.4	66.4 (81)	1.6 (0.8 – 3.4)	1.6 (0.7 – 3.4)^a^

^*^p < 0.05; ^**^p < 0.01.

^a^Adjusted OR for sex and age.

### Discriminative validity of the generic and condition specific OIDP score

No statistically significant differences occurred in the prevalence of impacts (64.5% versus 62.4%) and mean generic OIDP ADD scores (4.1 versus 2.8) between the hypodontia and non-hypodontia group. The mean generic OIDP ADD score increased with increasing severity of hypodontia and with anterior and upper anterior localisations of hypodontia, with effect sizes of 0.4, 0.4 and 0.5 respectively. The association was significant (p < 0.05) for upper anterior hypodontia. No significant association was found between severity or localisation of hypodontia and the prevalence of oral impacts, when using the generic OIDP scale (Table 4).

**Table 4:** Discriminative validity of generic OIDP: association of OIDP with presence or absence of hypodontia, the severity of hypodontia and anterior or upper anterior localisation of hypodontia

	**Mean OIDP (SD)**	**Effect size**	**OIDP > 0, % (n)**	**Unadjusted OR (95% CI)**	**Adjusted OR**^**a**^**(95% CI)**
***Presence of hypodontia***					
Non-hypodontia	2.8 (3.9)		62.4 (63)	1	1
Hypodontia	4.1 (6.0)	0.3	64.5 (40)	1.1 (0.6 – 2.1)	1.0 (0.5 – 2.0)
***Severity of hypodontia***					
Non-hypodontia	2.8 (3.9)		62.4 (63)	1	1
Mild (1–5 teeth missing)	3.5 (4.7)	0.2	64.5 (20)	1.1 (0.5 – 2.5)	1.1 (0.5 – 2.6)
Severe (≥ 6 teeth missing)	4.7 (7.0)	0.4	64.5 (20)	1.0 (0.7 – 1.6)	0.9 (0.6 – 1.4)
***All anterior hypodontia***					
Non-hypodontia	2.8 (3.9)		62.4 (63)	1	1
Anterior hypodontia^b^	4.8 (6.6)	0.4	74.4 (32)	1.8 (0.8 – 3.9)	1.6 (0.7 – 3.5)
Other hypodontia	2.5 (4.1)	0.1	42.1 (8)	0.7 (0.4 – 1.1)	0.6 (0.4 – 1.0)
***Upper anterior hypodontia***					
Non-hypodontia	2.8 (3.9)		62.4 (63)	1	1
Upper anterior hypodontia^c^	5.7 (7.3)^*^	0.5	75.0 (24)	1.8 (0.7 – 4.4)	1.6 (0.7 – 4.0)
Other hypodontia	2.4 (3.5)	0.1	53.3 (16)	0.8 (0.6 – 1.3)	0.8 (0.5 – 1.2)

^*^p < 0.05.

^a^Adjusted OR for sex and age.

^b^At least one tooth missing in the anterior segment.

^c^At least one tooth missing in the maxillary anterior segment.

A total of 30.6% of the hypodontia patients versus 9.9% of the non-hypodontia patients (p < 0.01) reported at least one CS oral impact (Table 5). A pattern of increasing CS OIDP scores occurred with anterior localisation (effect size 0.7, p < 0.01), increasing severity (effect size 0.8, p < 0.01) and upper anterior localisations of hypodontia (effect size 0.9, p < 0.01). The CS OIDP discriminated significantly between the following groups: non-hypodontia versus hypodontia, non-hypodontia versus severe hypodontia, non-hypodontia versus all anterior hypodontia and non-hypodontia versus upper anterior hypodontia, in adjusted regression analysis (Table 5).

**Table 5:** Discriminative validity of condition specific OIDP: association of condition specific OIDP and the presence or absence of hypodontia, the severity of hypodontia and anterior or upper anterior localisation of hypodontia

	**Mean OIDP (SD)**	**Effect size**	**OIDP > 0, % (n)**	**Unadjusted OR (95% CI)**	**Adjusted OR**^**a**^**(95% CI)**
***Presence of hypodontia***					
Non-hypodontia	0.3 (1.1)		9.9 (10)	1	1
Hypodontia	2.4 (5.5)^**^	0.6	30.6 (19)^**^	4.0 (1.7 – 9.4)	3.4 (1.4 – 8.0)
***Severity of hypodontia***					
Non-hypodontia	0.3 (1.1)		9.9 (10)	1	1
Mild (1–5 teeth missing)	1.4 (4.2)	0.4	16.1 (5)	1.8 (0.5 – 5.6)	1.6 (0.5 – 5.2)
Severe (≥ 6 teeth missing)	3.4 (6.6)^**^	0.8	45.2 (14)^**^	2.7 (1.7 – 4.4)	2.5 (1.5 – 4.3)
***All anterior hypodontia***					
Non-hypodontia	0.3 (1.1)		9.9 (10)	1	1
All anterior hypodontia^b^	3.0 (6.3)^**^	0.7	37.2 (16)^**^	5.4 (2.2 – 13.3)	4.8 (1.9 – 12.0)
Other hypodontia	1.1 (3.3)	0.4	15.8 (3)	1.3 (0.7 – 2.6)	1.2 (0.6 – 2.4)
***Upper anterior hypodontia***					
Non-hypodontia	0.3 (1.1)		9.9 (10)	1	1
Upper anterior hypodontia^c^	3.8 (7.1)^**^	0.9	43.8 (14)^**^	7.1 (2.7 – 18.4)	7.0 (2.6 – 18.7)
Other hypodontia	0.9 (2.7)	0.3	16.7 (5)	1.3 (0.8 – 2.4)	1.1 (0.6 – 2.1)

^**^p < 0.01.

^a^Adjusted OR for sex and age.

^b^At least one tooth missing in the anterior segment.

^c^At least one tooth missing in the maxillary anterior segment.

Among patients with hypodontia, no correlation occurred between the numbers of missing teeth and scores of the individual OIDP items, even when accounting for retained primary teeth (relative hypodontia). However, a significant correlation was found between anterior relative hypodontia and oral impacts on eating (rho = 0.29, p < 0.05) and speaking (rho = 0.34, p < 0.01), and also between anterior absolute hypodontia and oral impact on speaking (rho = 0.39, p < 0.01). Age was not correlated to the overall OIDP scores in the hypodontia group or the non-hypodontia group.

### Item specific generic and CS OIDP scores by clinical study group

Percentage and mean scores for the eight generic OIDP items according to clinical groups are shown in Table 6. Only with respect to emotional status, the prevalence of impacts as well as the mean impact score was significantly higher in the hypodontia than in the non-hypodontia group (p < 0.05). Although not statistically significant, the percentage and mean scores were consistently higher among hypodontia compared to non-hypodontia patients across the eight generic OIDP items.

**Table 6:** Prevalence of impacts and mean score for the eight generic OIDP items according to clinical group

	**Hypodontia (n = 61)**		**Non-hypodontia (n = 98)**	
	**% affected**	**Mean score (SD)**	**% affected**	**Mean score (SD)**
1. Eating	39.3	0.6 (1.0)	27.7	0.4 (0.8)
2. Speaking	19.7	0.5 (1.2)	16.0	0.3 (0.9)
3. Cleaning teeth	23.0	0.4 (0.8)	20.8	0.4 (0.9)
4. Sleeping and relaxing	9.8	0.2 (0.7)	13.0	0.2 (0.5)
5. Showing teeth	37.1	1.0 (1.5)	36.6	0.9 (1.4)
6. Emotional status	27.9^*^	0.6 (1.2)^*^	14.0	0.2 (0.7)
7. Social contact	19.7	0.5 (1.2)	11.9	0.2 (0.7)
8. Carrying out work	11.5	0.2 (0.8)	8.9	0.2 (0.6)

^*^p < 0.05.

Statistically significant differences between the clinical groups were found for the following CS OIDP items: speaking (p < 0.05), showing teeth, emotional status, social contact and carrying out work (all with p < 0.01) (Table 7).

**Table 7:** Prevalence of impacts and mean score for the eight condition specific OIDP items according to clinical group

	**Hypodontia (n = 61)**		**Non-hypodontia (n = 98)**	
	**% affected**	**Mean score (SD)**	**% affected**	**Mean score (SD)**
1. Eating	11.3	0.2 (0.8)	5.0	0.1 (0.5)
2. Speaking	9.7^*^	0.4 (1.1)^*^	2.0	0.1 (0.4)
3. Cleaning teeth	1.6	0.0 (0.1)	1.0	0.0 (0.2)
4. Sleeping and relaxing	1.6	0.1 (0.5)	1.0	0.0 (0.1)
5. Showing teeth	25.8^**^	0.7 (1.4)^**^	4.0	0.1 (0.4)
6. Emotional status	11.3^**^	0.4 (1.1)^**^	0.0	-
7. Social contact	14.5^**^	0.5 (1.2)^**^	2.0	0.0 (0.2)
8. Carrying out work	6.5^*^	0.2 (0.7)	1.0	0.0 (0.3)

^*^p < 0.05; ^**^p < 0.01.

## Discussion

This study confirms the theoretical expectation that the CS OIDP inventory was better able than the generic OIDP to discriminate between adolescents with and without hypodontia across various indicators of discriminative ability. Although the prevalence of oral impacts was always higher with the generic OIDP, the effect sizes for the generic OIDP were small to moderate with respect to severity, anterior localisation and upper anterior localisation of hypodontia, using the thresholds defined by Cohen [24]. In contrast, the corresponding effect sizes using the CS OIDP were moderate to large. The differences in overall scores and prevalence of impacts between groups with and without hypodontia, their corresponding effect sizes and adjusted ORs were always statistically significant with respect to CS OIDP (Table 5). Using the generic OIDP, the corresponding figures were, with one exception, not statistically significant (Table 4). The discriminative ability demonstrated in this study adds to the construct validity of the generic and CS OIDP. Moreover, the results indicate that patients with hypodontia attribute their oral impacts to small teeth, gaps between teeth and missing teeth, aspects clearly associated with hypodontia, suggesting a subjective treatment need in this group.

Consistent with the present results, Mbawalla et al. [25] concluded that the CS OIDP attributed to dental caries and malocclusion was better suited than its generic counterpart to support clinical indicators when estimating need for oral health care among Tanzanian adolescents. The CS OIDP was also found to discriminate better between groups with and without normative treatment need for dental caries, periodontal disease, malocclusion and dental injuries in Thai adolescents [26], as well as between groups with and without normative need for orthodontic treatment in Brazilian adolescents [27]. The present results are also in accordance with previous studies where generic and CS forms of OHRQoL measures were reported to perform differently [28-30]. Yet, only a few studies have compared the generic and CS forms of the same OHRQoL measure. This study compared, for the first time, generic and CS oral impacts of patients having hypodontia of different severity and localisation with the impacts of malocclusion patients having a normative need for orthodontic treatment similar to that of the patients with hypodontia.

The observed internal consistency reliability for the generic and CS OIDP in both study groups was above the level of 0.7 recommended by Cohen [24]. As reported in previous work [13], higher OIDP scores were associated with poor oral health perceptions and dissatisfaction with dental appearance, indicating satisfactory psychometric properties for the generic OIDP among Norwegian adolescents.

This study revealed a prevalence of generic and CS impacts in patients with hypodontia amounting to 64% and 30%, respectively. The corresponding rates in the non-hypodontia malocclusion group were 62% and 10%. The generic OIDP prevalence obtained among patients with and without hypodontia, is higher than that reported among similarly aged adolescents in Tanzania and UK [31,32], similar to that reported in Uganda [33] and lower than the prevalence rates reported from Thailand and Brazil [26,34]. Moreover, the generic OIDP prevalence rate is much higher than the normative OIDP values reported in the general Norwegian population above 16 years, where 18.2% reported oral impacts [13]. The quality of life impairment of the non-hypodontia group is consistent with several other studies reporting that malocclusion in itself gives rise to substantial impacts on psychosocial functioning [35,36]. This is also in accordance with Laing et al. [17] who examined a hypodontia group and a malocclusion control group, with similar normative treatment need, and found no difference in OHRQoL. The same study remarks that the reason for the findings is not known and that the cross-sectional design does not permit causality to be investigated. In the present study, differences in oral health related quality of life between the groups were obtained with the CS instrument only, indicating that a CS instrument is better able to discriminate between clinical groups compared to generic instruments. Studies comparing hypodontia patients to healthy controls [37], patients with a lesser degree of malocclusion (IOTN DHC 2–3) [18] or with normative data [20] report substantially more psychosocial impacts in the hypodontia group. Furthermore, studies investigating OHRQoL in patients with hypodontia, but without a control group, have reported oral impacts among all participants [19,38]. The control group in this study consisted of patients without hypodontia, but with a comparable degree of oral morbidity, rather than a Class I ideal occlusion. This might explain the poor observed discriminative ability of the generic OIDP inventory.

When examining the single generic OIDP item scores, problems with emotional status were significantly more prevalent in the hypodontia group than in the non-hypodontia group, indicating an emotional strain related to missing a substantial number of teeth (Table 6). The prevalence of CS impacts in the hypodontia group was highest for the impacts showing teeth, social contact, emotional status (Table 7). These three impacts are related to psychosocial functioning, emphasizing that missing teeth may carry a particular burden in the domain of normal socializing.

Surprisingly, there was no association between OIDP scores and either absolute or relative hypodontia. The mean relative hypodontia of 3.1 might indicate that the patient sample does not contain a large enough number of patients with a severe degree of hypodontia to detect associations related to the number of missing teeth. In addition, the adjacent teeth might have erupted into the gaps, camouflaging the congenital absence of one or more teeth. The positive association found between anterior relative hypodontia and the OIDP items eating and speaking may be explained by impeded bite function as well as reduced ability to pronounce sounds that require a complex coordination between palate, tongue and teeth. Laing et al. [17] also reported reduced functional abilities associated with relative hypodontia, and recommended retention of primary teeth when appropriate. The present results emphasise that the anterior segment is of particular importance in treatment planning. Remediation of the functional problems associated with anterior hypodontia may start at an early age by means of orthodontic mesialization, transplantation of premolars to the anterior segment, resin-bonded bridges and composite restorations of small permanent teeth or persisting primary teeth.

The hypodontia group reported worse self-rated oral health compared to others than the non-hypodontia group. However, the hypodontia patients were significantly more satisfied with their dental appearance. This suggests that although hypodontia is a major oral health issue, the patients may perceive it as a functional rather than an aesthetic problem when compared with severe malocclusions, which may strongly impact facial appearance. In addition, persisting primary teeth and composite restorations may mitigate the aesthetic consequences of hypodontia in the anterior segment. Compared to the non-hypodontia group, patients with hypodontia reported a significantly higher frequency of dental attendance, which can be ascribed to the need of attentive monitoring associated with these patients. Also, when treatment is commenced, patients with hypodontia are likely to need many visits for age-appropriate coordinated treatment. From an orthodontic point of view, the treatment for an isolated malocclusion is often predictable, with a set sequence being planned beforehand and usually smoothly implemented. In contrast, treatment for patients with hypodontia depends on several circumstances, such as the location of the missing teeth, the longevity of the primary teeth, the appropriate time for insertion of prosthetics and their durability. At the time of completing the questionnaire, most of the patients have not yet come to experience this reality, and are unaware of the extent of future treatment. With this in mind, a future study of young adults with a long experience of treatment for hypodontia would be of value to elucidate this difference.

## Conclusion

Norwegian patients with hypodontia and malocclusion report a substantial burden of oral impacts. The generic OIDP inventory did not discriminate between patients with and without hypodontia. The discriminative ability of the generic OIDP was independent of the particular indicators used to assess discriminative ability. The CS-OIDP measure attributed to hypodontia discriminated statistically significant between patients with and without hypodontia, and was related to severity and upper anterior localisation of hypodontia.

## Competing interests

The authors declare that they have no competing interests.

## Authors’ contributions

CH, KB and ANÅ designed the study protocol. CH and KB collected the OIDP data at the University of Oslo dental clinic. CH and ANÅ performed and interpreted the statistical analyses. CH drafted the manuscript, and all three authors read and approved the final manuscript.

## Pre-publication history

The pre-publication history for this paper can be accessed here:

http://www.biomedcentral.com/1472-6831/14/57/prepub
